# Supplementary material for: Atomic Layering Thermostable Antigen and Adjuvant (ALTA®) platform provides unique antigen delivery system through controlled release to improve immune response to vaccination
Source: bioRxiv. 2026 Jan 5:2026.01.05.697591. Preprint. [Version 1] doi: 10.64898/2026.01.05.697591 (PMC12803066; doi:10.64898/2026.01.05.697591)
Supplement: Supplement 1 [file media-1.pdf]

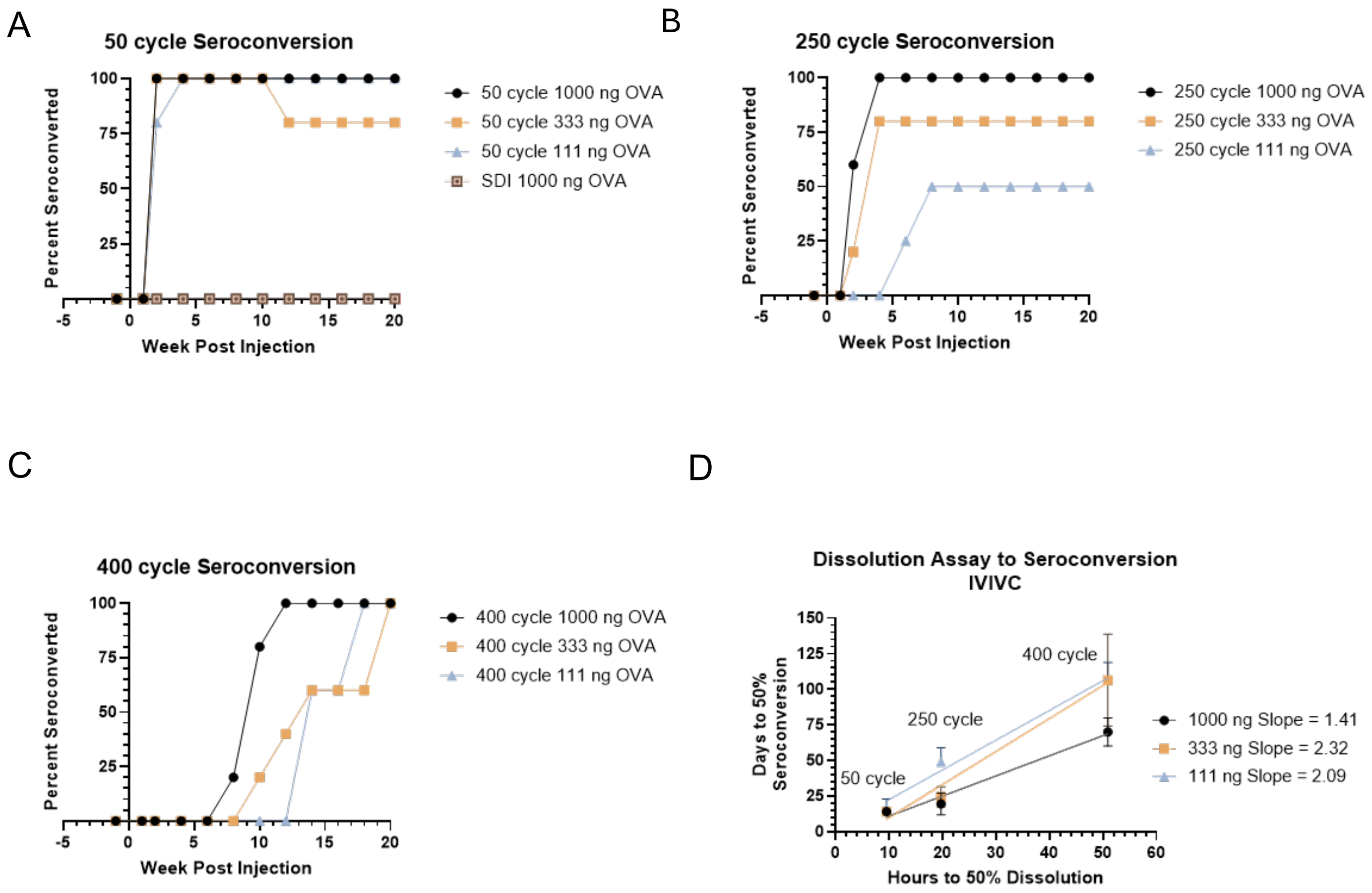

Supplementary Figure 1 – Delayed antibody responses depend upon coat thickness and dose administered

A-C. Anti-OVA IgG1 seroconversion percentage, indicating a 2 log-fold increase in anti-OVA IgG1 titers relative to pre-injection baseline, following vaccination with 50-cycle, (B) 250-cycle or (C) 400-cycle ALD coated powders at the indicated OVA doses.

D. Correlation of time to 50% particle dissolution in vitro and days to >50% seroconversion across treated animals in vivo at indicated OVA doses. Simple linear regression of data at each OVA dose shown, with slope of regression line indicated on plot.

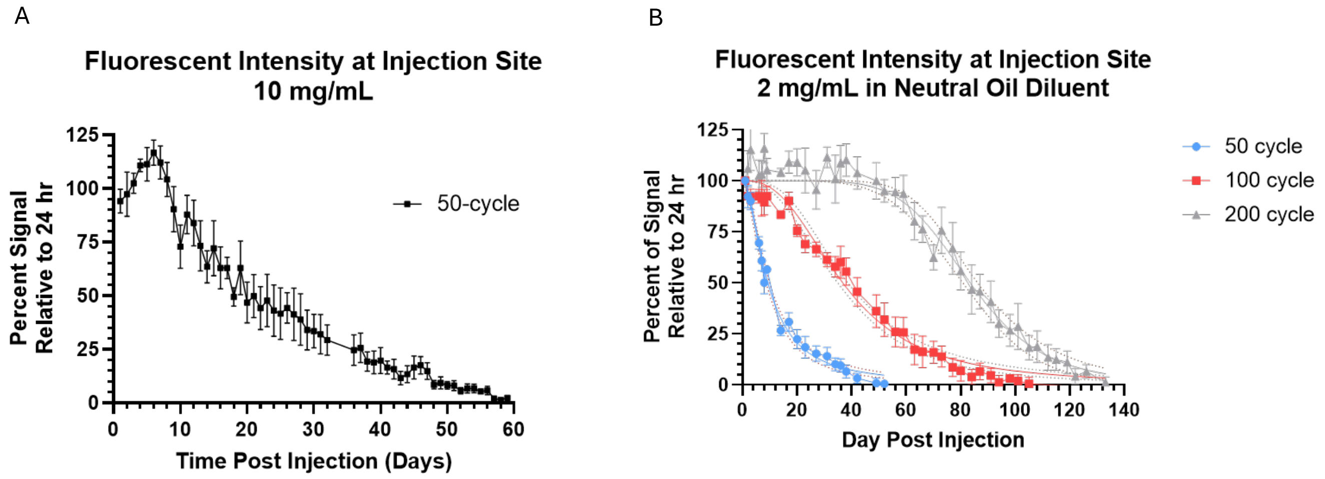

Supplementary Figure 2 - Increasing dose administered extends ALD-coated particle persistence at injection site

A. Mean +/- SEM percent of fluorescent radiant efficiency (p/s)/(μW/cm^2) relative to radiant efficiency at 24 hr timepoint measured at site of injection for 50-cycle ALD coated powder administered at 10 mg/mL (n=5 mice/group).

B. Mean +/- SEM percent of fluorescent radiant efficiency (p/s)/(μW/cm^2) relative to radiant efficiency at 24 hr timepoint measured at site of injection for 50-cycle, 100-cycle and 200-cycle ALD coated powder administered at 2 mg/mL using super refined sesame oil as the diluent for injection (n=5 mice/group).

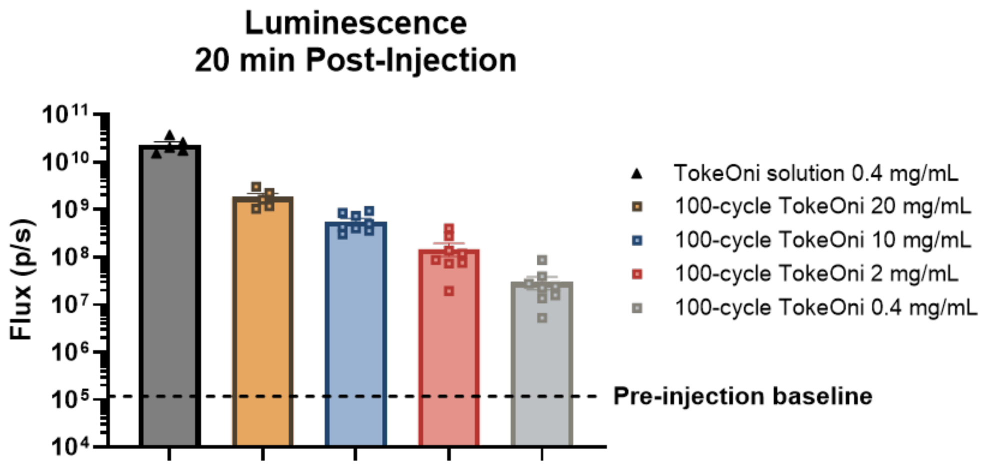

Supplementary Figure 3 – Activatable probe demonstrates presence of immediately soluble material in ALD coated powders

Total flux (p/s) measured at the site of injection 20 mins post administration of 100-cycle ALTA® powder containing TokeOni. A 0.4 mg/mL dose of TokeOni solution, matching the total TokeOni dose in 20 mg/mL 100-cycle ALTA® powder, was administered as a control.

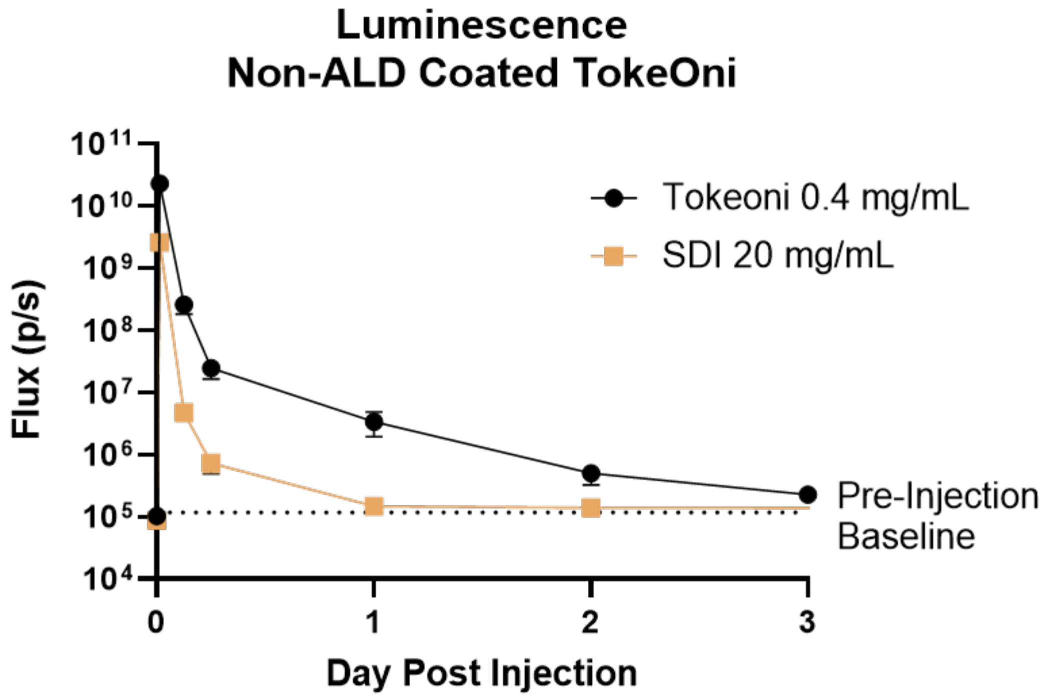

Supplementary Figure 4 – Duration of luminescent signal from liquid TokeOni solution and uncoated, reconstituted spray dried intermediate (SDI) TokeOni powder

Total flux (p/s) measured at the site of injection for 3 days following administration of 0.4 mg/mL TokeOni solution or 20 mg/mL uncoated SDI containing TokeOni (pre-cursor to ALD-coated powder) resuspended in sterile saline.

Sample	Area Size	%Percentage to SDI
SDI	53912	
Broken ALTA®	41478	78.7% (corrected for alumina%)

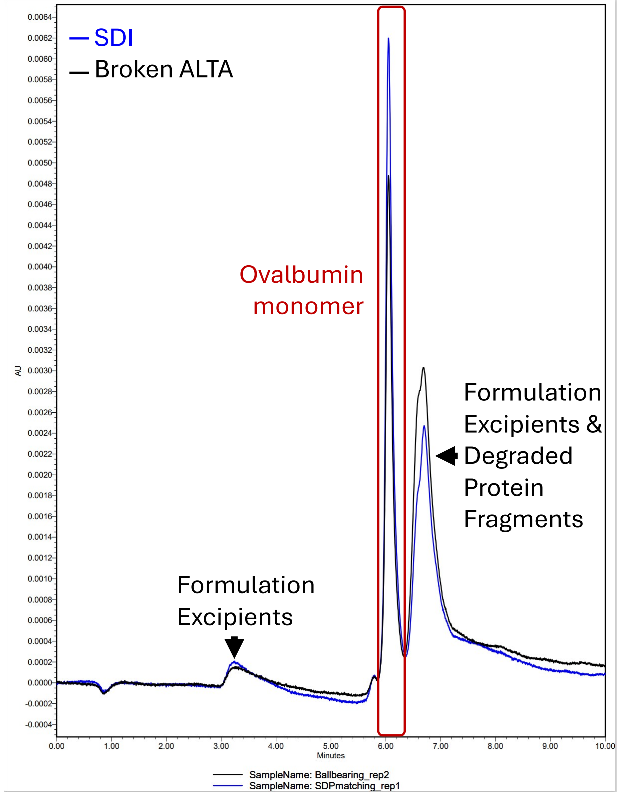

Supplementary Figure 5 – Uncoated SDI powder and broken 50-cycle ALTA® OVA powder chromatogram showed consistent peak patterns.

50-cycle ALTA® OVA was physically disrupted, then broken 50-cycle material or uncoated SDI powder was constituted in PBS/0.05% Tween-20 at 10 mg/mL. Size Exclusion Chromatography (SEC) was performed on the Waters ACQUITY UPLC Instrument.

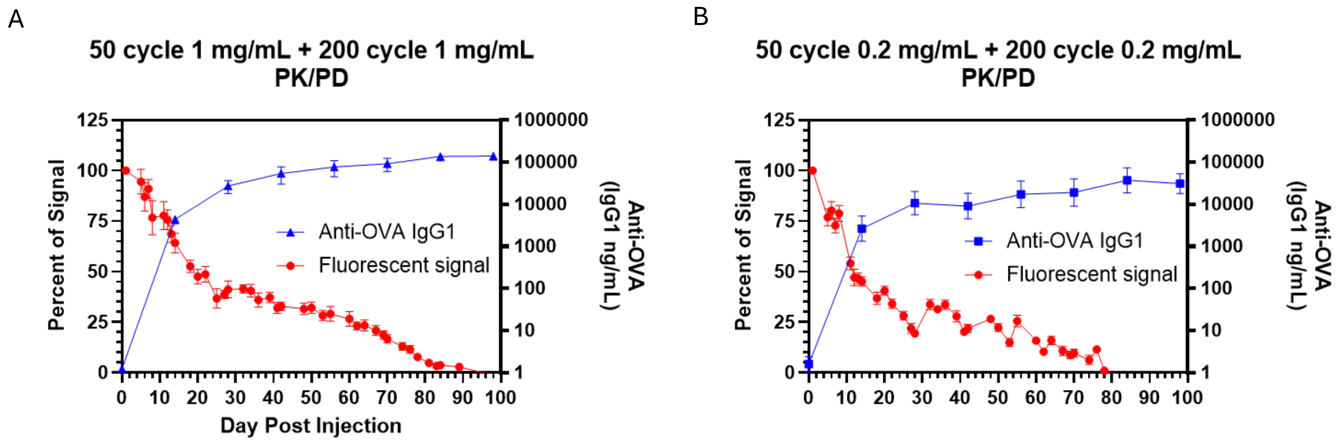

Supplementary Figure 6 – Mixed ALTA® products demonstrate release kinetics consistent with individual components, and elicit sustained antibody responses

A-B. Analysis of fluorescent signal at site of injection following administration of 50-cycle and 200-cycle ALTA® containing fluorescently-labeled OVA mixed at a 1:1 ratio dosed at total concentration of 2 mg/mL or (B) 0.4 mg/mL. Plot shows mean +/- SEM of percent of fluorescent radiant efficiency (p/s)/(μW/cm^2) relative to radiant efficiency at 24 hr timepoint measured at site of injection (n=5 mice/group) (red traces). Total anti-OVA IgG1 titers plotted as geometric mean +/- 95% confidence interval (n=5 mice/group) (blue traces).

	50-cycle	250-cycle	400- cycle
A (% Initial Release)	14%	2%	0.4%
B (Slope)	6.2	5.1	21.0
C (Hrs to Inflection)	9.5	29.2	50.9
D (% Full Release)	94%	98%	91%
% Alumina	2.3%	11.0%	15.7%

Supplementary Table 1 – In vitro dissolution assay results for ALTA® products tested in Supplementary Figure 1

In vitro dissolution assay 4PL fit parameters and alumina content (% w/w) of ALTA® products coated with 50, 250 or 400 ALD cycles.

	50-cycle 2 mg/mL	50-cycle 0.4 mg/mL	100-cycle 2 mg/mL	100-cycle 0.4 mg/mL	200-cycle 2 mg/mL	200-cycle 0.4 mg/mL
A - Bottom	0	0	0	0	0	0
D - Top	100	100	100	100	100	100
C - IC50 (Days to 50% signal loss)	15.31	13.38	31.20	27.87	77.16	63.18
B - Hill Slope	-3.226	-3.261	-2.408	-5.062	-4.893	-6.076
logIC50	1.185	1.126	1.494	1.445	1.887	1.801
R^2	0.8704	0.8002	0.7056	0.7899	0.6038	0.7933

Supplementary Table 2 – 4PL fit parameters of in vivo fluorescent imaging data shown in Figure 1

4PL analysis constrained to bottom = 0, top = 100. Column headers indicate ALD coat number and dose administered.

	50-cycle	100-cycle	200-cycle
A (% Initial Release)	19%	2%	0.8%
B (Slope)	2.7	9.7	16.6
C (Hrs to Inflection)	10.9	15.2	24.4
D (% Full Release)	104%	99%	102%
% Alumina	2.3%	11.0%	15.7%

Supplementary Table 3 – In vitro dissolution assay results for ALTA® products tested in Figure 1

In vitro dissolution assay 4PL fit parameters and alumina content (% w/w) of ALTA® products coated with 50, 100 or 200 ALD cycles.

Group	Product 1	Product 2	OVA dose Product 1	OVA dose Product 2	Administration Schedule
1	50-cycle ALTA®		200 ng		D0
2	100-cycle ALTA®		200 ng		D0
3	200-cycle ALTA®		200 ng		D0
4	50-cycle ALTA®	200-cycle ALTA®	100 ng	100 ng	D0
5	OVA-Alhydrogel 1:50 (D0)	OVA-Alhydrogel 1:50 (D28)	100 ng	100 ng	D0/D28
6	OVA-Alhydrogel 1:50 (D0)	OVA-Alhydrogel 1:50 (D49)	100 ng	100 ng	D0/D49
7	OVA-Alhydrogel 1:50	100-cycle ALTA®	100 ng	100 ng	D0
8	OVA-Alhydrogel 1:50	200-cycle ALTA®	100 ng	100 ng	D0

Supplementary Table 4 – In vivo experimental design related to Figure 4, comparing single administration of ALTA® OVA with two-dose liquid OVA-Alhydrogel formulations

Table indicates the products administered and the OVA dose contained within each product. Group 5 and Group 6 were given two separate injections of liquid OVA-Alhydrogel, with the second administration occurring on D28 or D49 post-prime, respectively. All other groups received a single administration on D0.
